# Case Management-based Collaborative Care Model Associated with improvement in neuropsychiatric outcomes in community-dwelling people living with dementia

**DOI:** 10.1186/s12877-023-04024-8

**Published:** 2023-05-31

**Authors:** Yu-Hsuan Hung, Wen-Fu Wang, Ming-Che Chang, Kai-Ming Jhang

**Affiliations:** 1 School of Medicine, Department of Medicine, Chung Shan Medical University, Taichung, Taiwan; 2 Department of Medical Education, Changhua Christian Hospital, Changhua, Taiwan; 3 Department of Neurology, Changhua Christian Hospital, Changhua, Taiwan; 4 Department of Nuclear Medicine, Changhua Christian Hospital, Changhua, Taiwan; 5 Present Address: No. 135 Nanxiao St, Changhua City, 500 Taiwan

**Keywords:** Behavioral and psychological symptoms of dementia (BPSD), Case management, Dementia collaborative care, Joint of Commission of Taiwan (JCT), Disease-Specific Care (DSC) certification program

## Abstract

**Background:**

This study aimed to explore the association between adherence of collaborative care model and short-term deterioration of BPSD after controlling patient and caregiver factors.

**Methods:**

This retrospective case–control study enrolled 276 participants who were newly diagnosed with dementia and BPSD. A dementia collaborative care team interviewed patients and caregivers to form a care plan and provided individualized education or social resource referrals. A multivariate logistic regression model with backward selection was used to test factors associated with BPSD deterioration, defined as worse neuropsychiatric inventory (NPI) scores 1 year after joining the care model.

**Results:**

Male sex (odds ratio [OR] = 0.45; 95% confidence interval [CI] = 0.25–0.84) and higher clinical dementia rating scale sum of boxes scores (CDR-SOB) (OR = 0.90; 95% CI = 0.83–0.98) were protective factors, whereas spouse caregivers and withdrawals from the care model (OR = 3.42; 95% CI = 1.28–9.15) were risk factors for BPSD deterioration.

**Conclusions:**

Our study showed that both patient and caregiver factors were associated with deterioration of BPSD. The case manager-centered dementia collaborative care model is beneficial for the management of BPSD. Healthcare systems may consider implementing a case management model in clinical dementia care practice.

## Introduction

As the proportion of people aged ≥ 65 reached the threshold level of 14% in Taiwan in 2018, which is referred to as “aged society”, the prevalence of dementia increased to 7.64% in older population aged > 65 years [1, 2]. Dementia affects cognitive function and usually accompanied with a series of neuropsychiatric symptoms (NPS) [3].

The clinical manifestations of behavioral and psychological symptoms of dementia (BPSD) vary. Behavioral symptoms usually identified by direct observation of the patients include agitation/aggression, disinhibition, hoarding, wandering, and aberrant motor activity, whereas psychological symptoms mainly assessed on the basis of interviews with patients and caregivers consist of delusions, hallucinations, dysphoria, anxiety, euphoria, and apathy [4]. The neuropsychiatric inventory (NPI) is one of the instruments used to systematically assess the presence and severity of BPSD [5]. The cumulative incidence of having one or more NPI-measured BPSD from the onset of cognitive symptoms was 80% [6], which could lead to a tough burden on both the patients and their caregivers [3, 7, 8].

Previous studies have investigated various factors of BPSD, which could be divided into patient, medical, and caregiver factors. In a Korean study, BPSD was found to be associated with the inability to live independently and disease severity in patients with Alzheimer’s disease (AD) [9]. Another study found that the frequencies of each BPSD varied among types of dementia, such as higher risk of delusions and hallucinations in dementia of Lewy bodies (DLB) and apathy and disinhibition in frontotemporal dementia compared to AD [10].

Concerning medical factors, the use of antipsychotics is common in the treatment of BPSD, and its effect on BPSD can vary among individuals [11]. A network meta-analysis suggested that physicians should individualize the assessment of antipsychotics because their results showed that no treatment options were effective and safe for patients with dementia [12]. While the adverse effects of antipsychotics could limit their effectiveness, studies also recommend careful ongoing observation and regular reassessment [13].

The significant influence of caregiver characteristics on BPSD severity should not be ignored. Previous studies have found that child and spouse caregivers tended to report different symptoms of BPSD, and specific symptoms such as delusions were correlated with spouse caregivers [14]. To ease the burden on caregivers, Taiwan’s government established the Long-Term Care Plan 2.0, which included dementia care services [15]. The dementia case management program was introduced to help dementia caregivers with easier access to medical resources such as individualized education, care plans, and social resources, including personal and professional services, transportation services, assistive devices, and respite care service [16, 17].

The presence of specific or clinically significant BPSD may predict disease progression, and elucidating the predictors associated with the improvement of BPSD is important [5, 18]. Although studies have discussed the effect of patient and caregiver factors on BPSD, relatively few have explored the use of long-term care resources and the efficacy of intervention by case managers. A randomized controlled trial reported that the total NPI score of patients with Alzheimer disease and caregivers’ stress and depression improved after intervention of collaborative care [19]. Another meta-analysis also showed significant improvement in patients’ behavior and caregivers’ burden, while the subgroup analysis found a case manager with nursing professionalism had greater positive effect on caregivers’ quality of life [20]. Thus, this study aimed to explore the association between adherence of collaborative care model and short-term deterioration of BPSD, defined as worse NPI scores 1 year after the first diagnosis of dementia with BPSD, after controlling patient and caregiver factors.

## Methods

### Study population

This retrospective case–control study was conducted at Changhua Christian Hospital in Central Taiwan from October 2015 to September 2021. Patients newly diagnosed with dementia were enrolled in the study. They underwent an initial assessment of cognitive function, BPSD, living status, care modes, and medications held by a dementia collaborative multidisciplinary care team. The care model consists of various professions, including physicians (neurologists, psychiatrists, gerontologists, and primary care physicians), psychologists, social workers, dieticians, occupational therapists, pharmacists, and nursing case managers. In addition to the initial interview, the care team arranged face-to-face evaluations, including living status, caring problems, caregiver burden, and preference to utilize long-term care resources every 6 months to renew the care needs of patients and their caregivers [17]. Nursing case managers followed the patients every month through phone calls to confirm if their needs were properly fitted. Patients and caregivers who refused or could not cooperate with the following face-to-face or phone evaluations were defined as withdrawing from the care model. Patients’ cognition and BPSD status were evaluated annually by clinical psychologists, regardless of withdrawal status. The care team passed the Disease- Specific Care (DSC) Certification Program for dementia held by the Joint Commission of Taiwan (JCT) in July 2021 [21]. The aforementioned information was recorded in the electronic charts of the nursing case managers.

This study was approved by the Institutional Review Board of Changhua Christian Hospital (CCH IRB 211,104). As all data required in this study were extracted from the electronic charts after deleting personalized information, informed consent was waived by the Institutional Review Board of Changhua Christian Hospital.

### Measurement of BPSD

We used the NPI to assess behavioral disturbances in patients with dementia [22]. Symptoms include delusions, hallucinations, dysphoria, anxiety, agitation/aggression, euphoria, disinhibition, irritability/lability, apathy, aberrant motor activity, nighttime behavior, and appetite disturbance. The assessment was performed at the initial assessment and 1 year later. Patients without NPS at the initial interview were excluded from the study. Participants with an elevation of the NPI total score 1 year after the initial evaluation (sum of 12 symptoms’ severity cross-frequency) were categorized into the deteriorated group, otherwise the non-deteriorated group. Patients with any one of the symptoms with a severity cross frequency ≥ 4 in the NPI were defined as having moderate-to-severe symptoms.

### Measurement of patient features

Data on patient age, sex, education level, clinical dementia rating scale sum of boxes (CDR-SOB) score, subtype of dementia, and walking ability were collected during the initial interview. CDR-SOB was used to quantify disease severity.

Dementia subtypes were classified according to different guidelines. The National Institute on Aging-Alzheimer’s Association (NIA-AA) corresponds to Alzheimer’s disease (AD), [23, 24] and the International Society for Vascular Behavioral and Cognitive Disorders (VASCOG) is used for vascular cognitive impairment (VCI) [25]. The Movement Disorder Society-Task force criteria and the fourth consensus report of the DLB Consortium is used for the diagnosis of Parkinson’s disease dementia (PDD) or DLB [26, 27]. Patients who were diagnosed with both AD and VCI are classified as having a mixed type. Patients with Lewy body disease (LBD) included those diagnosed with PDD or DLB.

### Measurement of caregiver features

Information on caregivers’ age, education level, relationship to the patients, cohabitant status, care mode, and usage of social resources were also collected during the interview. The care mode was classified into five levels: Mode 0 (the patient’s ADL was independent, and the caregiver only accompanied the patient), Mode 1 (care by a solo informal caregiver), Mode 2 (care by more than two caregivers that could include a foreign care worker), Mode 3 (care at different children’s homes alternately), and Mode 4 (care by a sole foreign care worker).

The utilization of long-term care resources was also recorded. According to the payment categorization in Long-Term Care Plan 2.0, long-term care resources were divided into four parts: personal and professional care (including in-home and community services such as home personal care or daycare services, home nursing, and home rehabilitation services), transportation services (shuttling from home to hospital), assistive devices and in-house barrier-free environment modification (the devices are suggested by therapists), and respite care (provided by personal care attendants to make caregivers rest for hours to days). In addition to the previous four parts, community aging care centers were set up extensively and offered places that provided congregate meals, general medical information, and exercise programs for healthy, pre-frail, and frail elderly people who still live in the community.

### Statistical analyses

R software (R Foundation for Statistical Computing) was used to generate all data in this study. The dependent variables included patient features and caregiver features, while the independent variable was deterioration of BPSD, defined as worsen NPI scores after 1 year. Differences between categorical data were calculated using Chi-squared test. Numerical data were analyzed using Student’s t-test. A multivariate logistic regression model with backward selection was used to test factors associated with worse BPSD. Statistical significance was determined when the p-value was less than 0.05.

## Results

This study enrolled 276 patients who were newly diagnosed with dementia and BPSD. Table 1 shows the baseline characteristics of the participants. The mean age of the deteriorated group was 79.02 years and that of the non-deteriorated group was 78.37. The CDR-SOB score of the deteriorated group was 4.70, which was lower than 5.63 in the non-deteriorated group. AD was the major type of dementia in both groups. The mean ages of the caregivers in the deteriorated and non-deteriorated group were 57.04 and 64.14 years, respectively. Most of the relationships between patients and caregivers were offspring. Most people living with dementia are cared for by sole informal caregiver. The percentages of utilization of long-term care resources and psychotropic drugs were comparable between groups. There was a significantly higher percentage of withdrawal from the care model in the deteriorated group (12.8% vs. 4.2%, *p* = 0.016). The deteriorated group had lower initial NPI total scores (6.82 ± 7.02 vs. 14.41 ± 14.60).

**Table 1:** Characteristics of patients and their caregivers between the deteriorated group and the non-deteriorated group

	Deteriorated N = 109	Non-deteriorated, N = 167	p- value*
Patient’s factor			
Age (SD)	79.02 (7.02)	78.37 (7.80)	0.485
Male (%)	35 (32.1%)	71 (42.5%)	0.107
Education: years (SD)	4.74 (4.26)	4.92 (4.21)	0.735
CDR-SOB (SD)	4.70 (3.11)	5.63 (3.93)	0.040
Diagnosis, type of dementia (%)			0.548
AD	65 (59.6%)	99 (59.3%)	
VCI	11 (10.1%)	24 (14.4%)	
Mixed ^**α**^	4 ( 3.7%)	5 (3.0%)	
LBD	18 (16.5%)	18 (10.8%)	
others	11 (10.1%)	21 (12.6%)	
Walking (%)			0.770
Free	63 (57.8%)	102 (61.1%)	
Assisted device	41 (37.6%)	56 (33.5%)	
Wheelchair	5 ( 4.6%)	9 ( 5.4%)	
Caregiver’s factor			
Age (SD)	57.04 (13.7)	64.16 (74.2)	0.323
Education: years (SD)	13.56 (12.5)	12.73 (12.5)	0.590
Relationship of caregivers (%)			0.591
Spouse	31 (28.4%)	50 (29.9%)	
Offspring	58 (53.2%)	94 (56.3%)	
Others	20 (18.3%)	23 (13.8%)	
Cohabitants (%)			0.561
Living alone	6 (5.5%)	11 (6.6%)	
Spouse only	25 (22.9%)	34 (20.4%)	
Children only	35 (32.1%)	46 (27.5%)	
Spouse and children	38 (34.9%)	60 (35.9%)	
Others	5 (4.6%)	16 ( 9.6%)	
Care mode (%) ^**β**^			0.870
0 1	21 (19.3%) 44 (40.4%)	27 (16.2%) 63 (37.7%)	
2	34 (31.2%)	58 (34.7%)	
3	1 ( 0.9%)	1 ( 0.6%)	
4	9 ( 8.3%)	18 (10.8%)	
Usage of long term care resources (%)			
Personal and professional care	15 (13.8%)	17 (10.2%)	0.474
Transportation	3 (2.8%)	6 (3.6%)	0.970
Assisted devices and home modification	8 (7.3%)	22 (13.2%)	0.185
Respite care	2 (1.8%)	1 (0.6%)	0.708
Community aging care centers	1 (0.9%)	2 (1.2%)	1.000
Medical factors			
Drug usage (%)			
Antipsychotics	43 (39.4%)	51 (30.5%)	0.162
Antidepressants	47 (43.1%)	59 (35.3%)	0.240
Sedatives	31 (28.4%)	45 (26.9%)	0.894
Withdrawal from care model (%) ^**γ**^	14 (12.8%)	7 (4.2%)	0.016
BPSD change			
Initial NPI (SD)	6.82 (7.02)	14.41 (14.60)	-
Followed NPI (SD)	18.72 (13.42)	5.98 (8.43)	-
NPI interval (year)	1.08 (0.36)	1.04 (0.38)	-

Footnotes: CDR-SOB, Clinical Dementia Rating Scale Sum of Boxes; NPI, Neuropsychiatric Inventory; AD, Alzheimer’s disease; VCI, vascular cognitive impairment; LBD, Lewy body disease

*From Student’s t test and Chi-square test for continuous and categorical variables, respectively

^**α“**^Mixed type” indicates mix AD and VCI

^**β**^ Care mode indicates that caregivers take care of people living with dementia most of the time. Mode 0 = ADL independent, caregivers only accompanied the subject; Mode 1 = care by sole informal caregiver; Mode 2 = care by more than two caregivers (can include foreign care workers); Mode 3 = care at different children’s homes alternately; Mode 4 = care by sole foreign care worker

^**γ**^ “Withdrawals” indicates either the patients or caregivers refused to keep contact with the hospital case managers

Table 2 shows the predictive factors for deterioration of BPSD using logistic regression with backward stepwise selection. Male sex and higher CDR-SOB were significant protective factors with odds ratios (OR) of 0.45 (95% CI = 0.25–0.84, *p* = 0.011) and 0.90 (95% CI = 0.83–0.98, *p* = 0.011). When the offspring were their main caregivers, BPSD tended to be non-deteriorated compared with spouse caregivers (OR = 0.30, 95% CI = 0.10–0.91, *p* = 0.034). Withdrawal from the care model increased 3.42 the risk of BPSD (95% CI = 1.28–9.15, *p* = 0.015).

**Table 2:** Multivariate logistic regression with backward selection to predict factors associated with deterioration of BPSD

	Odds Ratio	95% CI	p-value
**Patient’s factor**			
Age	1.04	[1.00;1.09]	0.067
Male	0.45	[0.25;0.84]	0.011
CDR_SOB	0.90	[0.83;0.98]	0.011
**Caregiver’s factor**			
Age	0.97	[0.94;1.01]	0.140
Relationship	Ref: Spouse		
Offspring	0.30	[0.10;0.91]	0.034
Others	0.31	[0.07;1.41]	0.130
Usage of long term care resources	Ref: without using		
Personal and professional care	1.85	[0.80;4.25]	0.149
Assisted devices and home modification	0.50	[0.19;1.30]	0.154
**Medical factors**			
Withdrawal from care model	3.42	[1.28;9.15]	0.015
Antipsychotic user	1.62	[0.94;2.78]	0.083

**Fig. 1:**
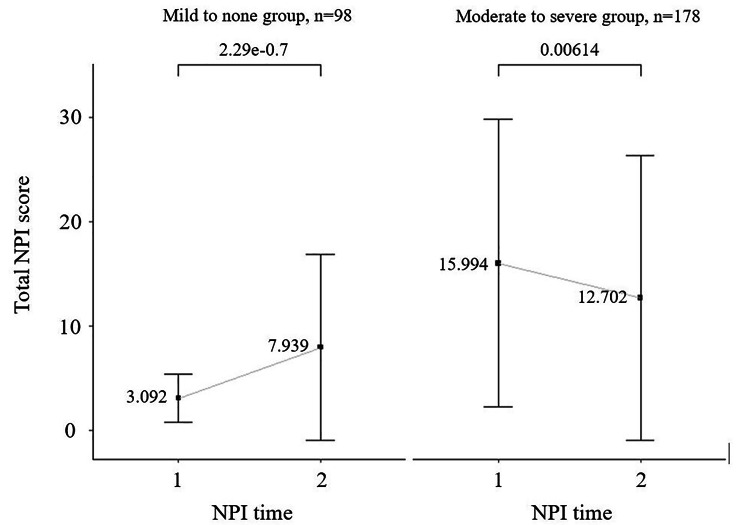
**Change in NPI scores by severity of BPSD** The moderate-to-severe group indicates that patients have any one of the symptoms’ severity cross-frequency ≧ 4 in the initial NPI.

Figure 1 shows the change in mean NPI total scores between the initial and follow-up interviews according to initial BPSD severity. A total of 276 patients were divided into mild to none group and moderate to severe group. The mild to none group (all symptoms’ severity cross-frequency < 4 in the initial NPI interview, n = 98) showed elevation of NPI scores (mean ± SD: 3.09 ± 2.31 and 7.94 ± 8.89 at first and followed-up NPI times, respectively). Adversely, the average score of the moderate-to-severe group significantly declined when the second interview was conducted (15.99 ± 13.75 and 12.70 ± 13.63 at first and second NPI times, respectively).

## Discussion

This study found that withdrawals from the collaborative care model carries a high risk for BPSD deterioration after controlling patient and caregiver factors. Female gender, milder disease severity, and spousal caregivers were also related to aggravation of BPSD.

Withdrawal from the collaborative care model is associated with higher risk for BPSD deterioration. The case managers of our care model played important roles in keeping contact with the patients, providing the caregivers with information on care services and communication skills toward BPSD, and suggesting that clinicians prescribe psychotropic medications in patients with severe NPS. A randomized clinical trial showed that collaborative care models lead by care managers improved both the quality of care and NPI scores [19]. A systematic review showed that primary care providers and case management partnership models improved the severity of NPS, caregiver burden, distress and mastery, and healthcare costs [28]. Studies also found that enrollment in collaborative care program could reduce BPSD severity and utilization of acute medical service [29]. The present study confirmed the effectiveness of case manager-centered collaborative care model on the severity of NPS.

Several cross-sectional studies have found that the severity of cognitive dysfunction is positively correlated with BPSD, particularly in patients with AD [9, 30, 31]. Longitudinal studies have found that the course of NPS varied highly and fluctuated [32, 33]. A 5-year cohort study found that cognitive decline was associated with higher NPI total scores and several NPI items [32]. When followed up for 12 years, more individual courses were observed, with most presenting as a single episode or relapsing instead of a stable condition [33]. The present result revealed that short-term changes in NPI scores in patients with milder dementia tend to deteriorate. The most likely explanation was that patients with higher CDR-SOB experienced more moderate-to-severe NPS, which was significantly associated with improved NPI scores (Fig. 1). Patients with a higher severity of NPS usually received comprehensive caregiver communication skills for BPSD and individualized non-pharmacological interventions by case managers of our collaborative care team. Medical treatment was introduced for patients with troublesome NPS. By contrast, in patients with mild disease and mild BPSD, the NPI score tended to increase during follow-up as the disease progressed.

The presentation of NPS may differ between sexes. Previous cohort study found that female patients had a higher risk of maintaining higher NPI scores for 3 consecutive years [34]. Another meta-analysis also showed that female sex was associated with a higher prevalence and greater severity of several NPS, such as depression, psychotic symptoms, and aberrant motor behavior [35]. While depressive symptoms were more frequent in female patients, male patients were prone to develop behavioral symptoms, such as verbal and physical abuse, wandering and delusions [36, 37]. The complicated factors contributing to sex differences included environmental factors, education level and genetic effect, and the aforementioned factors could give rise to interaction between hormones and clinical manifestations of BPSD [34].

Among caregiver factors, we found that spousal caregivers, as compared with offspring, were significant risk factors for BPSD deterioration. Lin et *al.* reported that a spouse’s primary caregiver was positively associated with the presence of delusions and the severity of anxiety and appetite/eating [14]. Previous studies have also indicated that patients’ NPI scores were with caregivers’ burden, and spouses, especially wives, usually reported higher levels of care burden than any other family members [38–40] Previous studies indicated that female caregivers were confronted with enormous social pressure, and they also tend to provide more caregiving assistance generally than male [41]. However, another study also found no significant association between female gender and number of tasks carried out after adjusting for possible confounders. Muhlichen et al. supposed that women tended to reflect the care burdens more intensely than men, and caregiver support programs were especially needed to relieve their burden [42]. On the other hand, spousal caregivers were found to have higher risk of depressive symptoms. In the cases of spousal and child caregivers, caregiving was viewed as an obligatory responsibility, which could cause these caregivers more likely to experience stress and depression [41].

Although not significant, antipsychotic use tended to increase the risk of BPSD deterioration in the present study. However, the effect of antipsychotics on BPSD remains controversial. In one Chinese study, failure to maintain antipsychotics could result in the recurrence of BPSD [43]. Conversely, a systemic review suggested that withdrawal of these medications does not lead to subsequent BPSD deterioration [44]. Keeping the use of antipsychotics in patients with higher NPI scores may prevent BPSD deterioration, whereas the others were recommended to taper medications to improve cognitive and psychomotor status [44, 45]. Antipsychotics were only reserved for patients with severe and persistent psychotic symptoms in our caring model, which may consist of a population with stable severe NPS who were prone to relapse of BPSD [33]. Long-term usage of antipsychotic polypharmacy remained controversial for patients with continued behavioral disturbances, as polypharmacy was perceived as a trigger for NPS [46]. Moreover, several antipsychotics and antidepressants had effects of acetylcholinesterase inhibitors (AChEI) and anticholinergics, respectively, and such polypharmacy interaction could increase the risk of cardiovascular events [47]. Lastly, although community aging care centers was effective to delay the decline of global function in dementia patients [48], use of long-term care services did not associate with short term BPSD changes in the present study. Long term effect of care resources on BPSD need further evaluation.

The strength of this study lies in the factors of people living with dementia and their caregivers, including caregiver features and the utilization of long-term care sources. Only a few studies have investigated the effect of dementia case management on BPSD severity, and the present study provides this perspective. However, this study had some limitations. First, the size of our study population was relatively small and was confined to a single hospital. Second, because the course of NPS usually fluctuates, the present study only reflects factors associated with short-term BPSD changes in individuals who are newly diagnosed with dementia. Long-term observations are needed to identify relevant risk factors and investigate the effectiveness of the care model on BPSD. Third, antipsychotic polypharmacy was not considered in the medical factor. However, there were less than 0.5% participants using more than two antipsychotics according to the regulations of Taiwan’s national health insurance.

## Conclusion

Male sex and higher CDR-SOB score decreased the severity of BPSD 1 year after the diagnosis of dementia, whereas spouse caregivers and withdrawals from the care model were associated with worse BPSD. The case manager-centered dementia collaborative care model is beneficial for the management of BPSD. Healthcare systems and insurers should consider implementing a case management model in clinical dementia care practice.

## Data Availability

The datasets used and/or analyzed during the current study are available from the corresponding author on reasonable request.
